# Association of anthropometric measures with all-cause and cause-specific mortality in US adults: revisiting the obesity paradox

**DOI:** 10.1186/s12889-024-18418-9

**Published:** 2024-04-01

**Authors:** Shan Li, Zhiqing Fu, Wei Zhang

**Affiliations:** 1 Department of Cardiology, Second Medical Center, Chinese People’s Liberation Army General Hospital, Beijing, 100853 China; 2 National Clinical Research Center for Geriatric Diseases, Chinese People’s Liberation Army General Hospital, Beijing, 100853 China; 3 Department of Outpatient, The Second Medical Center, Chinese People’s Liberation Army General Hospital, Beijing, 100853 China

**Keywords:** Central obesity, Overall obesity, Obesity paradox, Mortality

## Abstract

**Objective:**

Previous studies have shown that the obesity paradox exists in a variety of clinical settings, whereby obese individuals have lower mortality than their normal-weight counterparts. It remains unclear whether the association between obesity and mortality risk varies by anthropometric measures. The purpose of this study is to examine the association between various anthropometric measures and all-cause and cause-specific mortality in US adults.

**Methods:**

This cohort study included data from the National Health and Nutrition Examination Survey between 2009 and 2018, with a sample size of 28,353 individuals weighted to represent 231 million US adults. Anthropometric measurements were obtained by trained technicians using standardized methods. Mortality data were collected from the date of enrollment through December 31, 2019. Weighted Cox proportional hazards models, restricted cubic spline curves, and cumulative incidence analyses were performed.

**Results:**

A total of 2091 all-cause deaths, 606 cardiovascular deaths, 519 cancer deaths, and 966 other-cause deaths occurred during a median follow-up of 5.9 years. The association between body mass index (BMI) and mortality risk was inversely J-shaped, whereas the association between waist-to-height ratio (WHtR) and mortality risk was positively J-shaped. There was a progressive increase in the association between the WHtR category and mortality risk. Compared with the reference category of WHtR < 0.5, the estimated hazard ratio (HR) for all-cause mortality was 1.004 (95% confidence interval [CI] 1.001–1.006) for WHtR 0.50–0.59, 1.123 (95% CI 1.120–1.127) for WHtR 0.60–0.69, 1.591 (95% CI 1.584–1.598) for WHtR 0.70–0.79, and 2.214 (95% CI 2.200–2.228) for WHtR ≥ 0.8, respectively. Other anthropometric indices reflecting central obesity also showed that greater adiposity was associated with higher mortality.

**Conclusions:**

Anthropometric measures reflecting central obesity were independently and positively associated with mortality risk, eliminating the possibility of an obesity paradox.

**Graphical Abstract:**

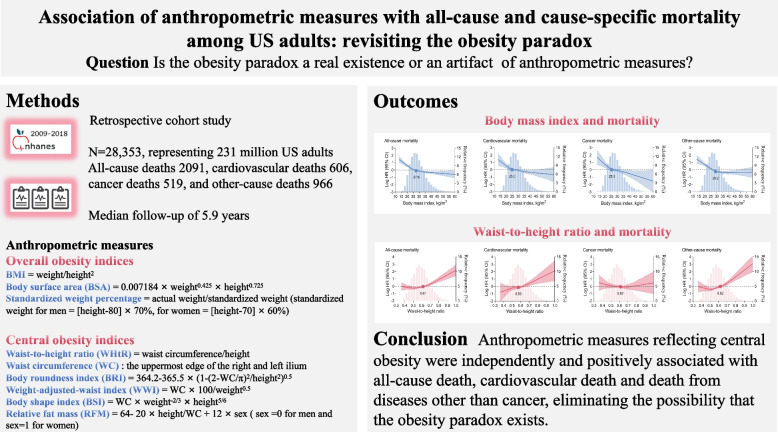

**Supplementary Information:**

The online version contains supplementary material available at 10.1186/s12889-024-18418-9.

## Introduction

Obesity is a growing public health concern, with the global prevalence predicted to reach 14% in men and 20% in women by 2030 [1]. In the United States, the prevalence of obesity will be as high as 47% in both men and women by that time [1]. The etiology of obesity is multifactorial and includes biology, genetics, socioeconomics, environmental factors, and access to healthcare resources [2]. Strong evidence suggests that obesity has deleterious effects on glucolipid homeostasis, blood pressure, systemic inflammation, and oxidative stress, thereby increasing the risk of various pathophysiological conditions such as diabetes, atherosclerosis, hypertension, musculoskeletal disorders, and certain cancers, as well as predisposing to premature death [3–7]. However, the existing literature reports that obese individuals have better survival than their normal-weight counterparts in a variety of clinical settings, a phenomenon known as the obesity-survival paradox [8–10]. This counterintuitive relationship may make it difficult to clarify the link between obesity and metabolic pathology and may send confusing messages to healthcare professionals and policymakers, potentially leading to hesitancy in controlling weight and adopting healthy lifestyles.

Several assessment tools have been used clinically to define excess body fat, including anthropometric, bioelectrical impedance analysis, densitometric and imaging-based methods [2]. Body mass index (BMI) is the most used anthropometric measure reflecting overall obesity. The obesity-survival paradox is typically documented using BMI as an evaluative indicator [8, 10, 11]. Epidemiological and genetic evidence suggests that the systemic metabolic risks of obesity depend not only on the amount of fat, but also on its distribution, and that central obesity, mainly the accumulation of abdominal or visceral fat, contributes to major cardiometabolic abnormalities and total mortality [12–15]. However, BMI has inherent limitations in defining adiposity because it can differentiate neither body compositions nor regional fat distribution, which weakens its credibility in predicting obesity-related metabolic risks and leads to heterogeneity or even conflicting epidemiologic relevance. Consequently, other anthropometric indices have been developed, including waist circumference, waist-to-height ratio (WHtR), waist-to-hip ratio (WHR), and body composition measured by dual-energy X-ray absorptiometry (DXA), which may be better surrogates for reflecting central obesity. However, the available evidence on the association between central obesity indices and mortality risk is not sufficient, so it remains unclear whether the obesity paradox reflected by BMI is a real existence or an artifact of anthropometric measures. It is of concern that the persistence of conflicting findings on the relationship between obesity and survival may misinterpret the considerable efforts toward weight control.

Therefore, we conducted this study in US adults from the 2009–2018 National Health and Nutrition Examination Survey (NHANES). The objectives of this study were to (i) examine the association of BMI and WHtR with all-cause and cause-specific mortality, using them as proxies for overall obesity and central obesity, respectively, (ii) characterize the association of other anthropometric indices reflecting overall obesity or central obesity with mortality risk, and (iii) attempt to explore the possible reasons for the discrepancy between anthropometric measures and the outcomes according to the correlation between anthropometric indices and DXA-based visceral fat measurements. We hypothesize that the obesity paradox may not be real but an artifact of anthropometric measures.

## Methods

### Study population

NHANES is a historical, nationally representative survey of the U.S. civilian noninstitutionalized population. The survey uses a multistage stratified probability cluster sampling design and incorporates participant weights to ensure accuracy in reflecting the demographics of the U.S. Census during the same time period [16]. We extracted data from five consecutive cycles of the NHANES database (2009–2010, 2011–2012, 2013–2014, 2015–2016, and 2017–2018). The study was conducted in accordance with the Declaration of Helsinki. All adult participants provided written informed consent, and all NHANES protocols were approved by the National Center for Health Statistics (NCHS) Ethics Review Board [17]. Of the 49,693 adults who participated in the 5 NHANES cycles, 21,340 were excluded because they were younger than 18 years (*n* = 19,341), pregnant (*n* = 247), had missing weight or height data (*n* = 1,596), had a BMI less than 10 kg/m^2^ or greater than 60 kg/m^2^ (*n* = 73), and had missing follow-up information (*n* = 83). Finally, 28,353 individuals were included in the analysis (Supplementary material Figure S1).

### Anthropometric measures

Participants wore disposable examination gowns and baseline weight, height and waist circumference were measured by trained health technicians to ensure methodological consistency. Waist circumference was measured at the uppermost edge of the right and left ilium, and waist circumference data were available for 26,998 individuals. The following anthropometric measures were examined, including overall obesity indices (BMI, body surface area [BSA], and standardized weight percentage) and central obesity indices (WHtR, waist circumference, body roundness index [BRI], weight-adjusted-waist index [WWI], relative fat mass [RFM], and body shape index [BSI]). The overall obesity indices were calculated based on weight and height, while the central obesity indices were calculated based on waist circumference and height (see Graphical abstract). According to the World Health Organization (WHO) criteria, individuals were divided into five BMI categories: underweight, < 18.5 kg/m^2^; normal weight, 18.5–24.9 kg/m^2^; overweight, 25–29.9 kg/m^2^; class I obesity, 30–34.9 kg/m^2^; and class II or III obesity, ≥ 35 kg/m^2^. For analyses using the WHtR, individuals were categorized as follows: < 0.50, 0.50–0.59, 0.60–0.69, 0.70–0.79, ≥ 0.80, with WHtR < 0.5 being the normal range. DXA-based visceral adipose tissue (VAT) measurements were available for 12,792 individuals, and VAT area and mass were measured at the L4 and L5 intervertebral spaces.

### Outcomes

The primary outcome was all-cause mortality, and the secondary outcomes were cardiovascular mortality, cancer mortality and other-cause mortality. Mortality data from the date of enrollment through December 31, 2019 were obtained by linking the NHANES dataset to death certificate records from the National Death Index (NDI) provided by the National Center for Health Statistics (NCHS) [18]. Cause-specific mortality was defined based on the recorded NCHS underlying classification of death (UCOD). Cardiovascular deaths were defined as deaths from heart disease and deaths from cerebrovascular disease. Cancer deaths were defined as deaths due to malignant neoplasms.

### Covariates

A wide range of covariates were considered, including age, sex, ethnicity, education, marital status, poverty income ratio, smoking status, alcohol consumption, systolic blood pressure, heart rate, BMI (for WHtR analyses) or waist circumference (for BMI analyses), atherosclerotic cardiovascular disease (ASCVD), diabetes mellitus, chronic obstructive pulmonary disease (COPD), cancer, aspirin, lipid-lowering drugs, hypoglycemic agents, and laboratory measurements (white blood cell count, hemoglobin, albumin, creatinine, urea nitrogen, glycohemoglobin, total cholesterol, and high-density lipoprotein cholesterol [HDL-C]). Demographic and health information was collected by experienced interviewers using a computer-assisted personal interview (CAPI) system and reviewed for completeness, consistency, and logicality to ensure data quality. Physical examinations were performed at a dedicated mobile examination center (MEC) using a uniform methodology and laboratory measurements were performed using the Beckman Coulter DxH 800 instrument for complete blood counts, and the Roche Cobas 6000 (c501 module) analyzer for standard biochemistry indices.

### Statistical analysis

All analyses were performed using R software (version 4.2.0) and EmpowerStats (X&Y Solutions, Inc., Boston, MA). Statistical significance was defined as a 2-tailed p-value < 0.05. Sample weights, stratification, and clustering were incorporated in all analyses to account for unequal selection and nonresponse probabilities. Baseline characteristics were expressed as means with standard deviations (SDs), medians with interquartile ranges (IQRs), or numbers with percentages and were compared by one-way analysis of variance, Kruskal–Wallis test, and chi-squared test. Data on covariates were more than 93% complete (Supplementary material Table S1). Missing values were imputed using chained equation multiple imputation (*n* = 5 data sets).

Restricted cubic spline curves based on Cox models were used to visualize the continuous association between BMI or WHtR and mortality. The inflection points of the mortality risk were estimated and the effect sizes before and after which were reported. Cox proportional hazards regression models were used to estimate hazard ratios (HRs) and 95% confidence intervals (CIs) of all-cause and cause-specific mortality for categorical and continuous WHtR. Proportional hazard assumptions were tested and confirmed by Schoenfeld's residual estimates and log(time) plots. Cumulative all-cause mortality for the BMI and WHtR groups was estimated using the Kaplan–Meier method, with the interval from the date of examination to the date of death or the end of follow-up as the time scale. Fine and Gray competing risk models were used for cause-specific mortality, with deaths from the two remaining causes as competing outcomes. The associations between other anthropometric indices and mortality were also visualized using restricted cubic spline curves. Linear regression fitting and Pearson's correlation coefficient were used to test the correlation between anthropometric indices and VAT measurements.

Sensitivity analyses were performed. First, we examined the association between other anthropometric indices (BSA, standardized weight percentage, waist circumference, BRI, WWI, RFM and BSI) as continuous variables and outcomes using the COX proportional hazards models. Second, we performed stratified analyses with subgroups of interest, including age, sex, ethnicity, and presence of diabetes mellitus. Third, individuals with less than 1 year of follow-up were excluded to minimize the potential bias for reverse causality. Fourth, complete case analyses were performed using only complete data for all covariates to assess whether missing data distorted the current results.

## Results

The sample included 28,353 individuals from the 2009–2018 NHANES data sets, weighted to represent 231 million US adults. During a median follow-up of 5.9 years, 2091 (7.4%) all-cause deaths, 606 (2.2%) cardiovascular deaths, 519 (1.8%) cancer deaths, and 966 (3.4%) other-cause deaths were recorded, respectively. BMI, BSA and standardized weight percentage were available for all individuals. Waist circumference was available for 95.2% of individuals, as were WHtR, BRI, WWI, RFM, and BSI. Of the total population, 32.1% were classified as overweight and 37.8% as obese, with a mean BMI of 29.0 (SD 6.8) kg/m^2^, while 83.0% had a WHtR outside the normal range of < 0.5, with a mean WHtR of 0.6 (SD 0.1). Individuals with higher BMI or WHtR were older, more likely to be non-Hispanic blacks, less likely to be current smokers, had a higher prevalence of ASCVD and diabetes mellitus, and had higher white blood cell and lower HDL-C levels. All-cause mortality increased progressively with increasing WHtR, while the opposite was present for BMI (Table 1 and Supplementary material Table S2).

**Table 1:** Baseline Characteristics of individuals by WHtR categories

Characteristics	WHtR					
	< 0.5	0.50- 0.59	0.60- 0.69	0.70–0.79	≥ 0.80	*P* value
**Unweighted**						
N (%)	4596 (17.0)	10,053 (37.3)	8306 (30.8)	3116 (11.5)	927 (3.4)	
All-cause mortality, n (%)	173 (3.8)	598 (5.9)	676 (8.1)	285 (9.1)	72 (7.8)	< 0.001
Cardiovascular mortality, n (%)	28 (0.6)	161 (1.6)	217 (2.6)	94 (3.0)	22 (2.4)	< 0.001
Cancer mortality, n (%)	48 (1.0)	174 (1.7)	169 (2.0)	69 (2.2)	14 (1.5)	< 0.001
Other mortality, n (%)	97 (2.1)	263 (2.6)	290 (3.5)	122 (3.9)	36 (3.9)	< 0.001
**Weighted**						
Age, years	35.3 ± 15.3	46.5 ± 16.5	51.4 ± 16.9	51.1 ± 16.8	49.0 ± 15.3	< 0.0001
Male, %	50.8	53.8	49.0	36.9	29.4	< 0.0001
Ethnicity, %						< 0.0001
Non-Hispanic White	65.6	66.1	64.1	66.0	63.2	
Non-Hispanic Black	13.2	9.3	11.0	13.3	15.7	
Hispanic	9.8	14.6	18.4	15.9	16.7	
Other	11.4	10.0	6.5	4.8	4.4	
Education level, %						< 0.0001
Under high school	12.5	14.6	17.7	17.3	16.9	
High school graduate	20.9	21.1	25.4	26.6	28.4	
Above high school	66.6	64.3	56.9	56.1	54.6	
Marital status, %						< 0.0001
Married/cohabiting	48.3	65.5	64.0	59.9	55.2	
Separated/divorced/widowed	9.7	16.5	20.9	23.6	23.5	
Never married/other	42.0	18.0	15.0	16.5	21.3	
Poverty income ratio (PIR)^a^	257 (103, 496)	296 (127, 500)	254 (113, 464)	222 (104, 416)	182 (89, 356)	< 0.0001
Smoking status, %						< 0.0001
Never smoker	64.1	57.5	55.0	53.0	56.5	
Former smoker	14.1	22.8	28.2	29.9	27.2	
Current smoker	21.8	19.8	16.8	17.2	16.3	
Alcohol consumption^b^, %						< 0.0001
Never	26.6	28.1	33.7	34.8	37.4	
Less than once a week	37.5	35.7	38.8	45.7	50.4	
More than once a week	35.9	36.1	27.5	19.4	12.2	
Body mass index, kg/m^2^	21.4 ± 2.3	26.2 ± 2.7	31.6 ± 3.3	38.3 ± 4.0	47.1 ± 5.1	< 0.0001
Waist circumference, cm	78.1 ± 5.9	92.8 ± 7.2	106.9 ± 7.6	121.9 ± 8.7	138.9 ± 10.2	< 0.0001
Heart rate, bpm	71.2 ± 11.6	71.3 ± 11.3	73.1 ± 11.7	74.8 ± 12.3	77.4 ± 12.7	< 0.0001
Systolic blood pressure, mmHg	114.8 ± 15.1	121.4 ± 17.0	125.6 ± 17.6	127.7 ± 17.8	129.8 ± 18.1	< 0.0001
Medical conditions						
ASCVD, %	2.2	6.0	9.6	10.6	13.0	< 0.0001
Diabetes mellitus, %	1.3	5.2	13.3	22.2	27.6	< 0.0001
COPD, %	2.5	3.3	5.4	7.3	10.4	< 0.0001
Cancer, %	5.3	9.8	12.1	13.0	12.1	< 0.0001
Laboratory measurement						
White blood cell, × 10^9^/L	6.6 ± 1.9	7.0 ± 2.0	7.5 ± 2.2	8.0 ± 2.2	8.6 ± 2.4	< 0.0001
Hemoglobin, g/dL	14.2 ± 1.4	14.3 ± 1.4	14.2 ± 1.5	14.0 ± 1.5	13.8 ± 1.5	< 0.0001
Albumin, g/L	44.4 ± 3.4	43.3 ± 3.1	42.2 ± 3.2	41.0 ± 3.1	39.4 ± 3.1	< 0.0001
Creatinine, umol/L	76.0 ± 19.7	78.5 ± 25.8	78.6 ± 27.8	76.6 ± 28.1	73.7 ± 23.1	< 0.0001
Urea nitrogen, mmol/L	4.5 ± 1.6	4.9 ± 1.7	5.1 ± 2.0	5.1 ± 2.2	5.0 ± 2.2	< 0.0001
Glycohemoglobin, %	5.3 ± 0.5	5.5 ± 0.7	5.8 ± 1.0	6.1 ± 1.2	6.3 ± 1.3	< 0.0001
Total cholesterol, mg/dL	177.5 ± 36.3	195.4 ± 41.0	195.2 ± 40.8	191.2 ± 40.0	184.4 ± 36.2	< 0.0001
HDL-C, mg/dL	61.4 ± 16.3	55.1 ± 16.5	49.5 ± 14.1	47.4 ± 12.7	46.1 ± 11.5	< 0.0001
Medications						
Aspirin, %	4.6	13.1	20.5	24.7	23.3	< 0.0001
Lipid-lowering drugs, %	3.4	14.3	24.2	26.9	25.0	< 0.0001
Hypoglycemic agents, %	1.1	4.5	12.0	21.6	26.1	< 0.0001

*WHtR* Waist-to-height ratio. *ASCVD* Atherosclerotic cardiovascular disease. *COPD* Chronic obstructive pulmonary disease. *HDL-C* High-density lipoprotein cholesterol

^a^The PIR is calculated by dividing family income by family size, year, and geographic location, based on the Department of Health and Human Services' poverty measure. ^b^A drink means at least 12 oz of beer, 5 oz of wine, or 1.5 oz of liquor

### Association between BMI or WHtR and mortality

**Fig. 1:**
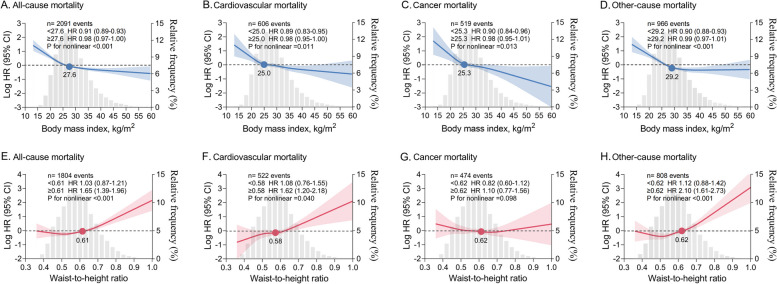
Nonlinear association between continuous BMI or WHtR and mortality. **A-D** BMI and mortality. **E**–**H** WHtR and mortality. HRs (solid lines) and 95% CIs (shaded areas) are based on weighted restricted cubic splines. The gray areas in the background show the distributions (histograms) of BMI or WHtR in the population. Solid dots represent risk inflection points for nonlinear associations. Effect size for per unit change in BMI (1 kg/m^2^) and WHtR (0.1) before and after the inflection point are shown separately. Models were adjusted for age, sex, ethnicity, waist circumference (for BMI analysis) or BMI (for WHtR analysis), education level, marital status, poverty income ratio, smoking status, alcohol consumption, systolic blood pressure, heart rate, ASCVD, diabetes mellitus, COPD, cancer, aspirin, lipid-lowering drugs, hypoglycemic agents, and laboratory measurements (white blood cell count, hemoglobin, albumin, creatinine, urea nitrogen, glycohemoglobin, total cholesterol, and HDL-C). ASCVD, atherosclerotic cardiovascular disease. COPD, chronic obstructive pulmonary disease. HDL-C, high-density lipoprotein cholesterol. HR, hazard ratio. CI, confidence interval

In restricted cubic spline analyses, there was an inversely J-shaped association between continuous BMI and mortality, with risk inflection points for all-cause mortality, cardiovascular mortality, cancer mortality, and other-cause mortality at BMIs of 27.6, 25.0, 25.3, and 29.2 kg/m^2^, respectively. The risk of death decreased sharply before the inflection point and remained almost constant thereafter. Conversely, there was a positively J-shaped association between continuous WHtR and mortality, with risk inflection points for all-cause mortality, cardiovascular mortality, and other-cause mortality occurring at WHtRs of 0.61, 0.58, and 0.62, respectively. The risk of death remained stable until the inflection point and then increased sharply and significantly. No significant association was found between WHtR and cancer death (Fig. 1). In Cox proportional hazards analyses, there was a gradual increase in the association between the WHtR category and all-cause mortality, cardiovascular mortality, and other-cause mortality. In addition, when WHtR was examined as a continuous variable, each 0.1 increase in WHtR was associated with a 36.8%, 43.7%, and 47.6% increase in all-cause, cardiovascular, and other-cause mortality, respectively (Table 2).

**Table 2:** Categorical or continuous WHtR and mortality by Cox proportional hazards model

**WHtR**	Model I HR (95% CI)	Model II HR (95% CI)	Model III HR (95% CI)
**All-cause mortality**			
< 0.50	reference	reference	reference
0.50–0.59	1.745 (1.742, 1.749)	0.772 (0.770, 0.774)	1.004 (1.001, 1.006)
0.60–0.69	2.645 (2.639, 2.651)	0.840 (0.839, 0.842)	1.123 (1.120, 1.127)
0.70–0.79	3.356 (3.348, 3.365)	1.190 (1.187, 1.193)	1.591 (1.584, 1.598)
≥ 0.80	3.429 (3.418, 3.441)	1.666 (1.660, 1.671)	2.214 (2.200, 2.228)
P for trend			0.007
Per-0.1 increase	1.410 (1.409, 1.411)	1.175 (1.175, 1.176)	1.368 (1.366, 1.371)
Per-SD increase	1.410 (1.410, 1.411)	1.175 (1.175, 1.176)	1.369 (1.366, 1.371)
**Cardiovascular mortality**			
< 0.50	reference	reference	reference
0.50–0.59	1.225 (1.218, 1.232)	1.501 (1.492, 1.510)	1.534 (1.524, 1.544)
0.60–0.69	1.541 (1.533, 1.550)	2.015 (2.001, 2.030)	2.045 (2.030, 2.060)
0.70–0.79	2.790 (2.773, 2.807)	3.470 (3.439, 3.501)	3.518 (3.486, 3.550)
≥ 0.80	3.082 (3.059, 3.106)	4.106 (4.054, 4.159)	3.590 (3.545, 3.635)
P for trend			< 0.001
Per-0.1 increase	1.594 (1.592, 1.595)	1.403 (1.401, 1.405)	1.437 (1.432, 1.442)
Per-SD increase	1.594 (1.593, 1.596)	1.403 (1.401, 1.405)	1.437 (1.432, 1.443)
**Cancer mortality**			
< 0.50	reference	reference	reference
0.50–0.59	0.756 (0.753, 0.759)	0.931 (0.928, 0.935)	0.960 (0.957, 0.964)
0.60–0.69	0.720 (0.717, 0.723)	0.878 (0.875, 0.882)	0.932 (0.929, 0.936)
0.70–0.79	0.773 (0.769, 0.776)	0.887 (0.884, 0.890)	0.972 (0.967, 0.977)
≥ 0.80	0.874 (0.868, 0.881)	0.916 (0.909, 0.923)	0.997 (0.989, 1.005)
P for trend			0.130
Per-0.1 increase	1.275 (1.273, 1.276)	1.012 (1.010, 1.013)	0.998 (0.996, 1.001)
Per-SD increase	1.275 (1.273, 1.277)	1.012 (1.010, 1.013)	0.998 (0.995, 1.001)
**Other-cause mortality**			
< 0.50	reference	reference	reference
0.50–0.59	1.413 (1.409, 1.417)	0.690 (0.688, 0.692)	1.038 (1.034, 1.042)
0.60–0.69	2.059 (2.053, 2.066)	0.750 (0.748, 0.753)	1.225 (1.219, 1.230)
0.70–0.79	2.645 (2.636, 2.654)	1.049 (1.045, 1.052)	1.903 (1.891, 1.915)
≥ 0.80	3.651 (3.636, 3.667)	1.858 (1.850, 1.866)	3.835 (3.801, 3.869)
P for trend			< 0.001
Per-0.1 increase	1.389 (1.388, 1.391)	1.171 (1.170, 1.172)	1.476 (1.472, 1.480)
Per-SD increase	1.390 (1.389, 1.391)	1.171 (1.170, 1.172)	1.477 (1.472, 1.482)

Model I, unadjusted. Model II, adjusted for age, sex, and ethnicity. Model III, additionally adjusted for BMI education level, marital status, poverty income ratio, smoking status, alcohol consumption, systolic blood pressure, heart rate, ASCVD Diabetes mellitus, COPD Cancer, aspirin, lipid-lowering drugs, hypoglycemic drugs, and laboratory measurements (white blood cell count, hemoglobin, albumin, creatinine, urea nitrogen, glycohemoglobin, total cholesterol, and HDL-C) based on model II. *WHtR* Waist-to-height ratio. *ASCVD* Atherosclerotic cardiovascular disease. *COPD* Chronic obstructive pulmonary disease. *HDL-C* High-density lipoprotein cholesterol. *HR* Hazard ratio. *CI* Confidence interval

### Cumulative mortality according to BMI or WHtR groups

**Fig. 2:**
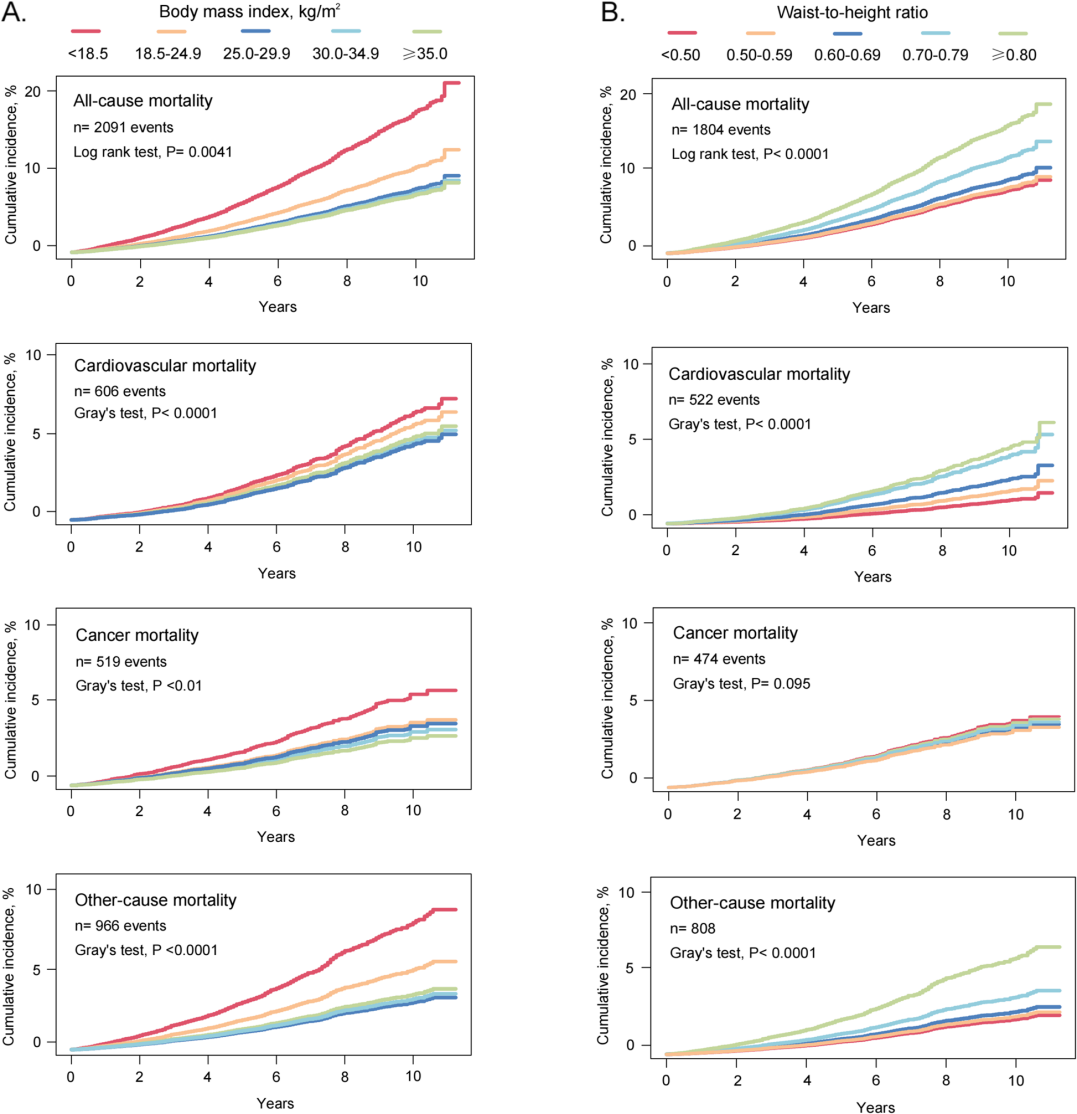
Cumulative mortality by BMI or WHtR groups. Cumulative incidence for mortality was estimated according to (**A**) BMI and (**B**) WHtR groups, and all-cause and cause-specific mortality was followed up to December 31, 2019. The Fine and Gray competing risk models were used for cause-specific mortality, with deaths from the remaining two causes as competing risks

The cumulative mortality curve showed a gradual decrease in mortality among groups with higher BMI. Specifically, the underweight group had the highest mortality, followed by the normal weight group, while the groups with overweight and obesity had the lowest mortality. Cumulative morbidity for WHtR showed the opposite pattern. Cumulative all-cause, cardiovascular, and other-cause deaths progressively increased in groups with incrementally higher WHtR, whereas there was no evidence that higher WHtR was associated with higher cumulative cancer mortality (Fig. 2).

### The concordance between BMI and WHtR

**Fig. 3:**
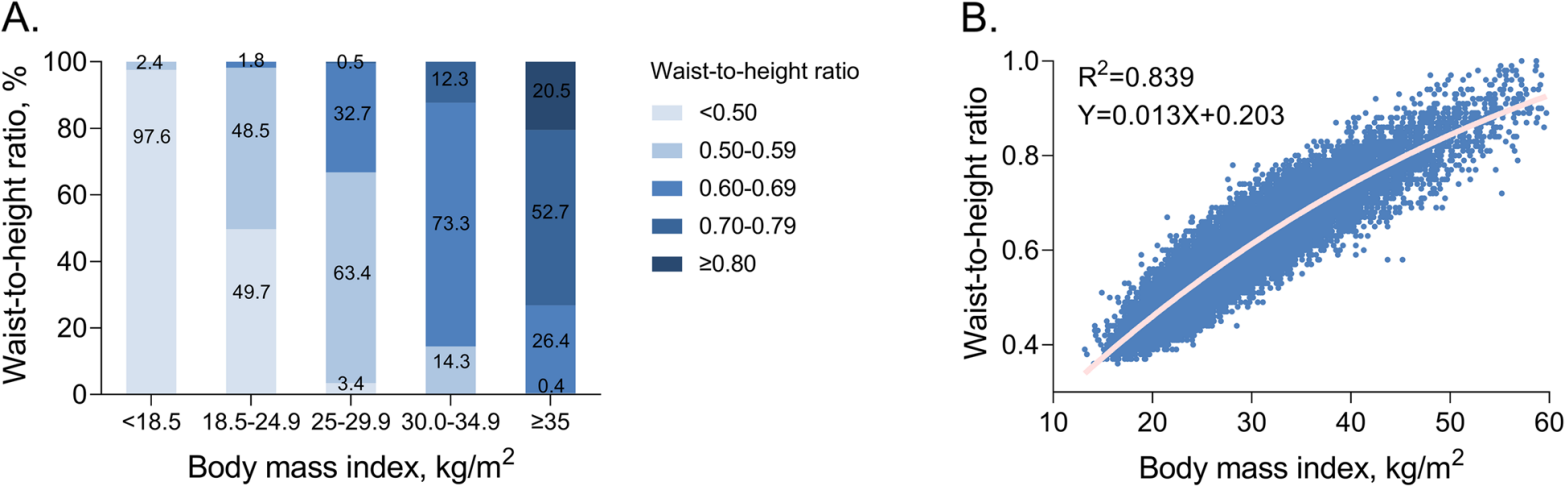
Correlation between BMI and WHtR categories. **A** WHtR percentage across BMI categories. **B** Correlation between BMI and WHtR categories

Although a strong correlation between BMI and WHtR was observed (*r*^2^ = 0.839), there were significant differences in agreement between subcategories. Specifically, 97.6% (489 out of 501) of underweight individuals had WHtRs within the normal range, whereas less than half of normal-weight individuals (49.7%, 3807 out of 7653) had WHtRs < 0.5, with the remaining half having WHtRs between 0.5 and 0.7. The concordance between BMI and WHtR was quite high among overweight and obese individuals, most of whom had WHtRs above the normal range (Fig. 3).

### Correlation between anthropometric indices and VAT measurements

**Fig. 4:**
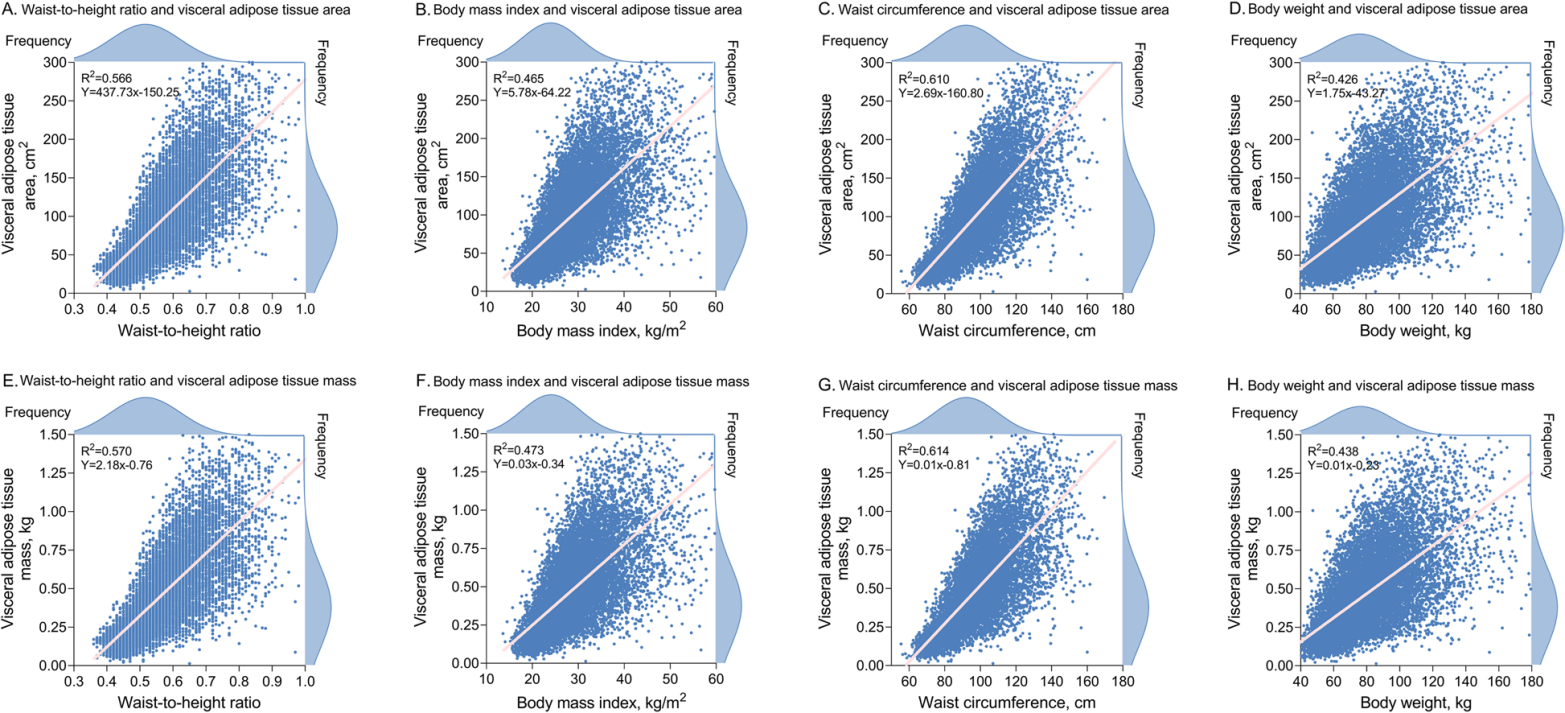
Correlation between anthropometric indices and visceral adipose tissue measurements Pink lines show linear regressions of anthropometric indices on visceral adipose area (**A**-**D**) and linear regressions of anthropometric indices on visceral adipose mass (**E**–**H**)

In addition to BMI and WHtR, we also examined the correlation of body weight and waist circumference with VAT, as the latter two are the basis for calculating the overall and central obesity indices, respectively. Overall, Pearson's correlations between anthropometric indices and VAT measures were modest. However, the correlations between VAT measures and WHtR or waist circumference were stronger than those with BMI or body weight, ranging from *r*^2^ = 0.566–0.614 for WHtR and waist circumference to *r*^2^ = 0.426–0.473 for BMI and body weight (Fig. 4).

### Other anthropometric indices and mortality

**Fig. 5:**
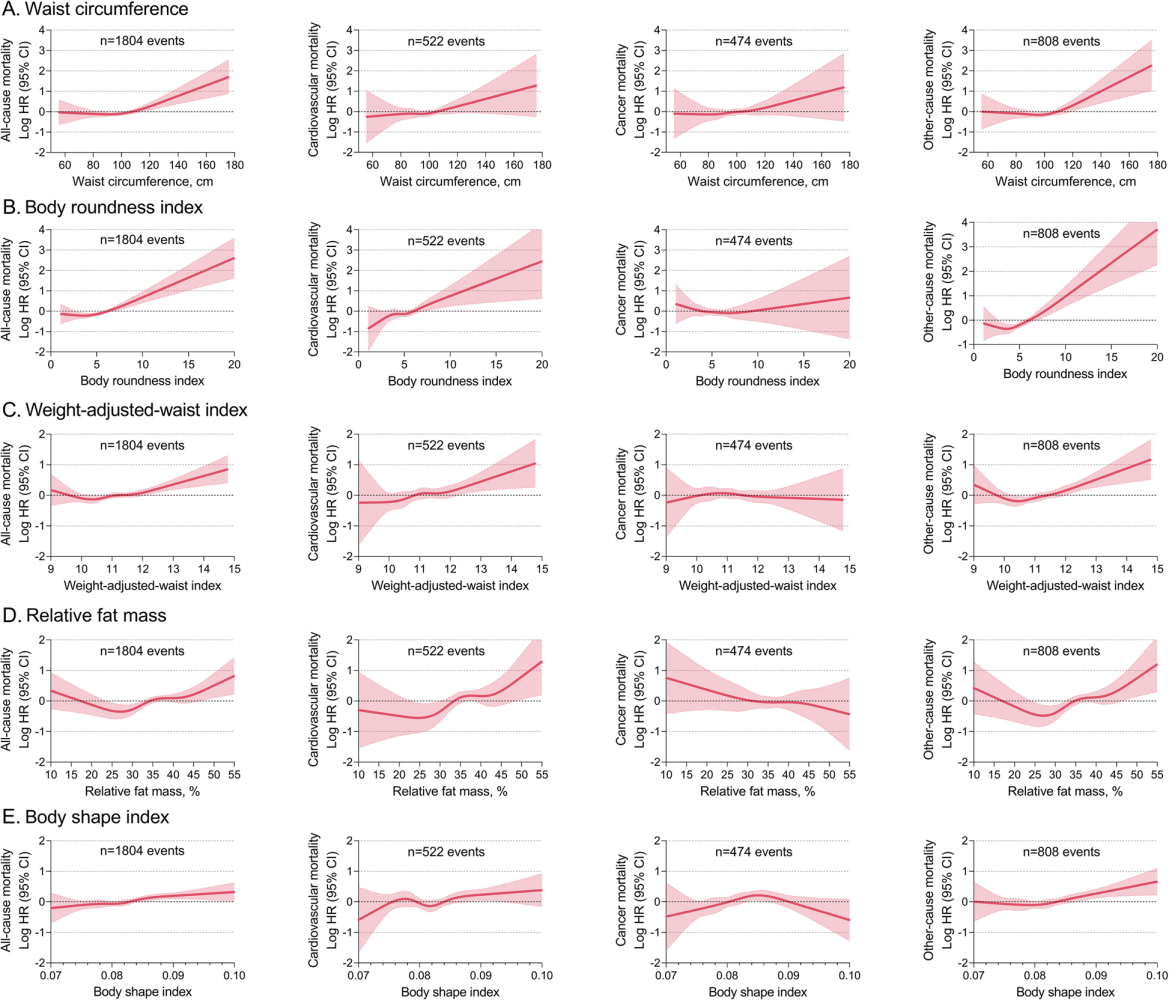
Nonlinear association between other central obesity indices and mortality HRs (solid lines) and 95% CIs (shaded areas) are based on weighted restricted cubic splines. The models were adjusted for age, sex, ethnicity, BMI, education, marital status, poverty income ratio, smoking status, alcohol consumption, systolic blood pressure, heart rate, ASCVD, diabetes mellitus, COPD, cancer, aspirin, lipid-lowering drugs, hypoglycemic agents, and laboratory measurements (white blood cell count, hemoglobin, albumin, creatinine, urea nitrogen, glycohemoglobin, total cholesterol, and HDL-C). ASCVD, atherosclerotic cardiovascular disease. COPD, chronic obstructive pulmonary disease. HDL-C, high-density lipoprotein cholesterol. HR, hazard ratio. CI, confidence interval

Other central obesity indices (waist circumference, BRI, WWI, RFM and BSI) also had positively J-shaped associations with all-cause, cardiovascular, and other-cause mortality, but no significant associations with cancer mortality (Fig. 5). Other overall obesity indices (BSA and standardized body weight percentage) had inversely J-shaped associations with mortality, whereas the association between BSA and cancer death did not appear to be significant (Supplementary material Figure S2).

### Sensitivity analysis

The results of the sensitivity analyses were generally consistent with those of the primary analyses. First, using the COX proportional hazards models, other central obesity indices (waist circumference, BRI, WWI, RFM and BSI) were positively associated with all-cause, cardiovascular, and other-cause mortality. In contrast, other overall obesity indices (BSA and standardized body weight percentage) were negatively associated with mortality (Supplementary material Table S3). Second, in subgroups stratified by age, sex, ethnicity, and diabetes, the association pattern between continuous BMI or WHtR and mortality was consistent with the primary analysis (Supplementary material Figures S3-S6). Third, exclusion of individuals with less than 1 year of follow-up did not substantially change the results (Supplementary material Figure S7). Fourth, a complete case analysis showed that missing data did not distort the current findings (Supplementary material Figure S8).

## Discussion

In a large nationally representative cohort of US adults, we examined the association between various anthropometric measures and all-cause and cause-specific mortality with a maximum follow-up of 11.3 years. We found that BMI and WHtR had diametrically opposite associations with mortality risk. The association between BMI and mortality was inversely J-shaped, whereas the association between WHtR and mortality was positively J-shaped. Other anthropometric indices of overall obesity also suggested a negative association between obesity and mortality, whereas none of the central obesity indices supported such a counterintuitive relationship. The current findings suggest that the obesity paradox may be an artifact of anthropometric measures, and that central obesity indices were independently and positively associated with all-cause death, cardiovascular death, and death from diseases other than cancer, eliminating the possibility that the obesity paradox exists.

The obesity-survival paradox has been previously reported in studies of critically ill patients, the elderly, and the general population [8-10]. A meta-analysis involving 218,532 patients with cardiovascular disease also demonstrated that total mortality was lower in overweight and obese patients than in normal weight patients, with a hazard ratio of approximately 0.70 [19]. Using BMI as an anthropometric indicator, we replicated this counterintuitive association through an inversely J-shaped pattern, whereby the risk of death decreased gradually within the initial units of BMI and then reached a plateau. All the nadirs of mortality risk were in the overweight range, lending further credence that a higher BMI may be protective for survival. In addition, when other indices of overall obesity were examined, the results were similar to those of the BMI, suggesting that overall obesity indices derived from weight-based calculations are consistent in estimating mortality risk.

Previous findings on the association between central obesity indices and adverse outcomes have been heterogeneous, with some studies reporting J-shaped or monotonic positive associations and others showing negative or null associations [20–22]. Methodologically, some of these studies did not address the hard endpoint of mortality, some focused on all-cause mortality and lacked data on cause-specific mortality, and some analyzed only a single anthropometric index. Our results provide evidence on these unaddressed issues. We found a positively J-shaped association between WHtR and all-cause, cardiovascular, and other-cause mortality, independent of BMI. The risk inflection point occurred around a WHtR of 0.6, slightly above the currently recommended threshold of 0.5, with slight change in the risk of death until the inflection point, followed by a sharp and linear increase. This positive association pattern was consistently observed for other anthropometric indices of central obesity, including waist circumference, BRI, WWI, RFM and BSI, albeit slightly attenuated for RFM and BSI. The current findings are in accordance with those of several recent large studies revealing an independent positive association between central obesity indices and adverse outcomes (e.g., premature mortality, heart failure hospitalization, cardiometabolic risk), some of which used a Mendelian randomized design to infer causality [23–25]. We did not find a substantial association between WHtR and cancer mortality. The association between obesity and cancer incidence and mortality varies by cancer site. The International Agency for Research on Cancer (IARC) has reported seven cancers for which there is compelling evidence of a dose–response relationship with obesity, including cancers of esophagus, colorectum, pancreas, cardia, liver, gallbladder, and kidney [26]. However, the three most common cancers in the current cohort were breast (15.4%), prostate (15.3%), and skin cancers (14.8%), which accounted for nearly half of individuals with cancers. This may explain our inability to find a clear association between obesity and cancer death in this nationally representative population.

The divergent association pattern may be due to differences in population-level risk classification using different anthropometric measures. In the present study, despite a strong linear correlation between BMI and WHtR, the consideration of WHtR resulted in a significant reclassification of individuals with normal weight. Only half of the normal weight individuals fell within the normal range of WHtR, suggesting that the pathophysiological milieu of the remaining half may be overlooked. There was less misclassification of overweight or obese individuals, with 98% having a WHtR greater than 0.5. A minority of overweight or obese individuals have a normal WHtR, which may be due to increased muscle mass rather than fat accumulation. These individuals, known as metabolically healthy obese (MHO), have been previously documented [27]. Our findings suggest that BMI is not sufficient to identify the high-risk phenotype for central obesity as defined by the WHtR, especially in those with normal weight (underestimation of risk). Previous evidence has also shown that high-risk characteristics for central obesity include a higher ratio of visceral-to-subcutaneous adipose tissue, a larger waist circumference, and a higher ratio of waist circumference to hip or leg circumference [28], which can be captured by central obesity indices rather than BMI alone.

Epidemiological and genetic evidence has shown that the regional distribution of fat may be more important than its absolute mass in predicting obesity-related metabolic risk [14, 25, 29]. Computed tomography (CT) and magnetic resonance imaging (MRI) allow accurate quantification of the body compositions at each level, thereby identifying subcutaneous adipose tissue (e.g., gluteal and thigh fat) and VAT (e.g., intra-abdominal and ectopic fat) [30, 31]. However, CT involves ionizing radiation and MRI is time consuming, both are expensive and require specially trained personnel to perform. DXA serves as a viable alternative with low radiation exposure and low cost, and has been validated by CT and MRI in identifying the high-risk metabolic phenotype [32, 33]. We found moderate correlations between anthropometric indices and VAT measurements based on DXA, it is in line with the expectation that an anthropometric indicator that can only make a rough estimate of fat distribution. Specifically, WHtR and waist circumference had stronger correlations with VAT measurements than BMI, whereas body weight had the weakest correlation, and the correlation coefficients were in agreement with previous studies [32]. Mechanistically, subcutaneous adipose tissue plays a critical role in energy storage and thermoregulation, and when its storage capacity is saturated, adipotoxic VAT deposition occurs. VAT exerts adipocyte biological effects through increased secretion of pro-inflammatory adipokines and decreased secretion of anti-inflammatory adipocytokines [2, 34]. Consequently, VAT creates an atherogenic, diabetogenic, and inflammatory milieu leading to downstream metabolic dysregulation and cardiovascular damage [35]. Because routine measurement of VAT may be impractical, the use of alternative anthropometric indicators as simple estimates in clinical practice is promising. The stronger correlation between WHtR and VAT measurements compared to BMI may partly explain why WHtR provides a better estimate for adverse outcomes.

The growing obesity epidemic is associated with substantial mortality, morbidity, and health care expenditures. Therefore, obesity has been included in the global targets for the control of non-communicable diseases (NCD) [36]. Based on current research, we have several considerations. First, it is imperative to implement comprehensive and effective prevention strategies that focus on promoting healthy lifestyles and controlling excessive weight gain. However, the existence of the obesity-survival paradox may cause confusion and hesitation among the public and policy makers. Our findings suggest that the obesity paradox may be an artifact of anthropometric measures rather than an actual biological advantage of excess fat storage, which dispels concerns that being overweight or obese improves survival over being normal weight. Second, susceptibility to obesity-related metabolic risk may be mediated by visceral fat, and anthropometric measures of central obesity provide independent and additive information beyond BMI in characterizing adverse risk. A growing number of obesity professional societies have recommended that central obesity indices (waist circumference or WHtR) should be routinely used alongside BMI for the stratification and management of obesity [15, 37]. Third, accurate assessment of obesity requires consideration of the validity, feasibility, and standardization of assessment indicators. Measuring waist circumference alone is inadequate because it does not consider the effect of height, which is significantly and inversely associated with health risks such as cardiovascular disease and cancer [38, 39]. Waist-hip ratio is a valid indicator for considering both VAT and lower-body subcutaneous adipose tissue. However, hip circumference is less readily available, making waist-to-hip ratio less practical. The WHtR corrects waist circumference by height, normalizing the threshold to 0.5, regardless of gender, age, and ethnicity. This simplifies the health message to the notion that waist circumference should not exceed half of one's height, offering a more feasible and pragmatic measure for both health professionals and the general population. Finally, further research should focus on whether the adoption of these anthropometric metrics can meaningfully enhance risk prediction algorithms beyond traditional measurements, and whether these anthropometric indicators can serve as valid targets for risk reduction.

## Strengths and limitations

We applied the weights in each of the models to account for oversampling of minority groups, survey nonresponse, and post-stratification adjustments. Baseline anthropometric measurements were completed by trained technicians rather than self-reported height and weight, thereby mitigating anthropometric bias. A wide range of covariates were adjusted to maximize consideration of confounding factors. Mortality events were provided by the NCHS using an enhanced linkage algorithm that allowed for 98.5% matching accuracy. Several limitations should be noted. First, due to the inherent limitations of the observational study, we cannot prove a causality relationship. Second, despite our efforts to comprehensively adjust for confounders, residual confounders may still exist. However, the statistical E-values for the associations between WHtR > 0.8 and all-cause, cardiovascular, and other-cause mortality were 3.83, 6.64 and 7.14, respectively, implying that the unmeasured confounders should have an association with the exposure (WHtR > 0.8) and outcome (mortality) comparable to these values to negate the current results. Third, although we excluded individuals with less than 1 year of follow-up to rule out reverse causation and obtain similar risk estimates, complete elimination of reverse causation cannot be achieved because individuals may survive with a disease for a longer period before succumbing to it. Fourth, DXA data were available for only half of the cohort, so the correlation between anthropometric indices and VAT measures should be interpreted with caution.

## Conclusion

Anthropometric measures reflecting central obesity were independently and positively associated with mortality risk, eliminating the possibility of an obesity paradox. WHtR provides additional information beyond BMI and can be used as a valid anthropometric indicator for physical examination screening.

### Supplementary Information

**Supplementary Material 1.**

## Data Availability

The NHANES data that support the findings of this study are available on the Center for Disease Control web site (https://www.cdc.gov/nchs/nhanes/).
