# Supplementary material for: Association of anthropometric measures with all-cause and cause-specific mortality in US adults: revisiting the obesity paradox
Source: BMC Public Health. 2024 Apr 1;24:929. doi: 10.1186/s12889-024-18418-9 (PMC10983763; doi:10.1186/s12889-024-18418-9)
Supplement: Supplementary file 1 — Supplementary Material 1. [file 12889_2024_18418_MOESM1_ESM.docx]

Supplementary Material

**Association of anthropometric measures with all-cause and cause-specific mortality in US adults: revisiting the obesity paradox**

Shan Li^1,2^, Zhiqing Fu^1,2^, Wei Zhang^2,3^

^1^Department of Cardiology, Second Medical Center, Chinese People’s Liberation Army General Hospital, Beijing 100853, China.

^2^National Clinical Research Center for Geriatric Diseases, Chinese People’s Liberation Army General Hospital, Beijing 100853, China

^3^Department of Outpatient, The Second Medical Center, Chinese People’s Liberation Army General Hospital, Beijing 100853, China

**Legends**

**Figure S1. Individual inclusion flowchart**

**Table S1. Missing data**

**Table S2. Baseline characteristics of individuals by BMI categories**

**Figure S2. Nonlinear association between other overall obesity indices and mortality**

**Table S3. Association between other anthropometric indices and mortality**

**Figure S3. Association of BMI and WHtR with mortality, stratified by age**

**Figure S4. Association of BMI and WHtR with mortality, stratified by sex**

**Figure S5. Association of BMI and WHtR with mortality, stratified by ethnicity**

**Figure S6. Association of BMI and WHtR with mortality, stratified by presence of diabetes**

**Figure S7. Association of BMI and WHtR with mortality, excluding individuals with less than 1 year of follow-up**

**Figure S8. Association of BMI and WHtR with mortality, complete case analysis**

**Figure S1. Individual inclusion flowchart**

**NHANES 2009-2018**

**n=49,693**

2009-2010 n=10,537

2011-2012 n=9,756

2013-2014 n=10,175

2015-2016 n=9,971

2017-2018 n=9,254

**21,340 were excluded**

<18years n=19,341

Pregnant n=247

Without weight or height n=1,596

BMI <10 or >60 kg/m^2^ n=73

Without follow-up data n=83

**28,353 individuals were included**

(26,998 individuals had waist circumference data)

**Table S1. Missing data**

| **Missing data (Unweighted)** | **BMI analysis** | **WHtR analysis** |
| --- | --- | --- |
| Waist circumference | 1350 (4.8%) | 0 |
| Heart rate | 1026 (3.6%) | 729 (2.7%) |
| Systolic blood pressure | 1170 (4.1%) | 855 (3.2%) |
| Laboratory measurement |  |  |
| White blood cell | 1341 (4.7%) | 1061 (3.9%) |
| Hemoglobin | 1330 (4.7%) | 1050 (3.9%) |
| Albumin | 1763 (6.2%) | 1446 (5.4%) |
| Creatinine | 1782 (6.3%) | 1460 (5.4%) |
| Urea nitrogen | 1768 (6.2%) | 1449 (5.4%) |
| Glycohemoglobin | 1339 (4.7%) | 1055 (3.9%) |
| Total [cholesterol](https://wwwn.cdc.gov/Nchs/Nhanes/2017-2018/BIOPRO_J.htm#LBXSCH) | 1693 (6.0%) | 1379 (5.1%) |
| High-density lipoprotein cholesterol | 1701 (6.0%) | 1385 (5.1%) |

**Table S2. Baseline characteristics of individuals by BMI categories**

| **Characteristics** | **Body mass index, kg/m^2^** | | | | | |
| --- | --- | --- | --- | --- | --- | --- |
|  | **< 18.5** | **18.5- 24.9** | **25.0- 29.9** | **30.0- 34.9** | **≥ 35.0** | **P value** |
| **Unweighted** |  |  |  |  |  |  |
| N (%) | 528 (1.9) | 8006 (28.2) | 9104 (32.1) | 5905 (20.8) | 4810 (17.0) |  |
| All-cause mortality, n (%) | 62 (11.7) | 590 (7.4) | 668 (7.3) | 438 (7.4) | 333 (6.9) | 0.003 |
| Cardiovascular mortality, n (%) | 10 (1.9) | 153 (1.9) | 201 (2.2) | 141 (2.4) | 101 (2.1) | 0.389 |
| Cancer mortality, n (%) | 16 (3.0) | 134 (1.7) | 173 (1.9) | 122 (2.1) | 74 (1.5) | 0.046 |
| Other mortality, n (%) | 36 (6.8) | 303 (3.8) | 294 (3.2) | 175 (3.0) | 158 (3.3) | <0.001 |
| **Weighted** |  |  |  |  |  |  |
| Age, years | 39.1 ± 19.3 | 43.4 ± 18.5 | 48.6 ± 17.3 | 49.0 ± 16.6 | 47.1 ± 16.1 | <0.0001 |
| Male, % | 35.0 | 43.9 | 55.6 | 53.0 | 40.0 | <0.0001 |
| Ethnicity, % |  |  |  |  |  | <0.0001 |
| Non-Hispanic White | 64.7 | 66.6 | 65.5 | 64.3 | 62.5 |  |
| Non-Hispanic Black | 12.1 | 9.4 | 9.7 | 12.2 | 16.7 |  |
| Hispanic | 8.4 | 11.1 | 16.6 | 17.5 | 15.9 |  |
| Other | 14.8 | 12.9 | 8.2 | 6.0 | 4.8 |  |
| Education level, % |  |  |  |  |  | <0.0001 |
| Under high school | 17.8 | 14.8 | 16.6 | 16.4 | 15.3 |  |
| High school graduate | 25.6 | 21.0 | 22.4 | 24.4 | 26.4 |  |
| Above high school | 56.7 | 64.3 | 61.0 | 59.2 | 58.3 |  |
| Marital status, % |  |  |  |  |  | <0.0001 |
| Married/cohabiting | 42.6 | 55.3 | 65.5 | 62.8 | 59.7 |  |
| Separated/divorced/widowed | 16.4 | 15.5 | 17.8 | 20.2 | 20.0 |  |
| Never married/other | 41.0 | 29.2 | 16.7 | 17.0 | 20.2 |  |
| Poverty income ratio (PIR)* | 188 (101, 372) | 306 (145, 500) | 314 (152, 500) | 297 (148, 500) | 247 (126, 446) | <0.0001 |
| Smoking status, % |  |  |  |  |  | <0.0001 |
| Never smoker | 56.4 | 60.0 | 56.7 | 55.7 | 56.9 |  |
| Former smoker | 12.7 | 18.4 | 25.5 | 26.5 | 26.8 |  |
| Current smoker | 31.0 | 21.6 | 17.7 | 17.8 | 16.3 |  |
| Alcohol consumption**, % |  |  |  |  |  | <0.0001 |
| Never | 39.4 | 30.6 | 31.7 | 32.7 | 34.1 |  |
| Less than once a week | 33.6 | 34.9 | 34.6 | 38.9 | 47.3 |  |
| More than once a week | 27.0 | 34.5 | 33.6 | 28.4 | 18.6 |  |
| Waist circumference, cm | 70.7 ± 4.7 | 82.9 ± 7.2 | 96.5 ± 7.2 | 107.7 ± 7.7 | 123.8 ± 12.2 | <0.0001 |
| Heart rate, bpm | 74.0 ± 13.5 | 71.5 ± 11.6 | 71.2 ± 11.4 | 72.7 ± 11.2 | 75.7 ± 12.4 | <0.0001 |
| Systolic blood pressure, mmHg | 115.7 ± 20.9 | 118.8 ± 18.0 | 122.8 ± 17.3 | 124.5 ± 16.6 | 127.0 ± 17.1 | <0.0001 |
| Medical conditions |  |  |  |  |  |  |
| ASCVD, % | 6.6 | 5.6 | 7.3 | 9.8 | 9.3 | <0.0001 |
| Diabetes mellitus, % | 2.2 | 3.8 | 8.1 | 13.3 | 19.5 | <0.0001 |
| COPD, % | 8.7 | 3.8 | 4.0 | 4.4 | 6.6 | <0.0001 |
| Cancer, % | 8.5 | 9.5 | 10.8 | 10.3 | 10.7 | 0.0357 |
| Laboratory measurement |  |  |  |  |  |  |
| White blood cell, ×10^9^/L | 7.0 ± 2.1 | 6.8 ± 2.0 | 7.1 ± 2.0 | 7.4 ± 2.1 | 8.1 ± 2.3 | <0.0001 |
| Hemoglobin, g/dL | 14.0 ± 1.4 | 14.0 ± 1.4 | 14.3 ± 1.4 | 14.3 ± 1.5 | 14.0 ± 1.5 | <0.0001 |
| Albumin, g/L | 44.0 ± 3.9 | 43.6 ± 3.3 | 43.1 ± 3.2 | 42.3 ± 3.2 | 40.8 ± 3.3 | <0.0001 |
| Creatinine, umol/L | 71.5 ± 34.9 | 75.9 ± 27.4 | 79.5 ± 24.0 | 79.6 ± 26.0 | 76.7 ± 29.0 | <0.0001 |
| Urea nitrogen, mmol/L | 4.3 ± 2.0 | 4.8 ± 1.8 | 5.1 ± 1.9 | 5.1 ± 2.0 | 4.9 ± 2.1 | <0.0001 |
| Glycohemoglobin, % | 5.3 ± 0.5 | 5.4 ± 0.7 | 5.6 ± 0.8 | 5.8 ± 1.0 | 6.0 ± 1.2 | <0.0001 |
| Total [cholesterol](https://wwwn.cdc.gov/Nchs/Nhanes/2017-2018/BIOPRO_J.htm#LBXSCH), mg/dL | 177.8 ± 36.5 | 185.5 ± 39.5 | 195.5 ± 41.6 | 195.1 ± 40.7 | 189.1 ± 38.6 | <0.0001 |
| HDL-C, mg/dL | 63.3 ± 16.5 | 60.8 ± 16.8 | 53.0 ± 15.4 | 49.0 ± 13.9 | 46.6 ± 12.3 | <0.0001 |
| Medications |  |  |  |  |  |  |
| Aspirin, % | 9.1 | 10.0 | 16.8 | 19.2 | 19.0 | <0.0001 |
| Lipid-lowering drugs, % | 5.2 | 10.3 | 18.1 | 22.6 | 21.9 | <0.0001 |
| Hypoglycemic agents, % | 1.8 | 3.2 | 7.2 | 12.2 | 18.5 | <0.0001 |

*The PIR is calculated by dividing family income by family size, year, and geographic location, based on the Department of Health and Human Services' poverty measure. **A drink means at least 12 ounces of beer, 5 ounces of wine, or 1.5 ounces of liquor. ASCVD, atherosclerotic cardiovascular disease. COPD, chronic obstructive pulmonary disease. HDL-C, high-density lipoprotein cholesterol.

**Figure S2. Nonlinear association between other overall obesity indices and mortality**

Body surface area (BSA)

**
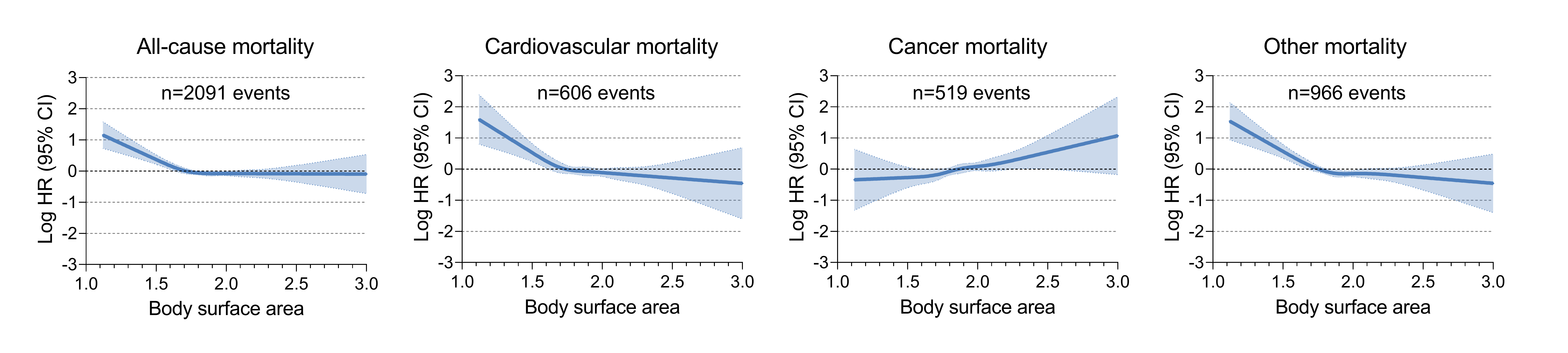
**

Standardized weight percentage

**
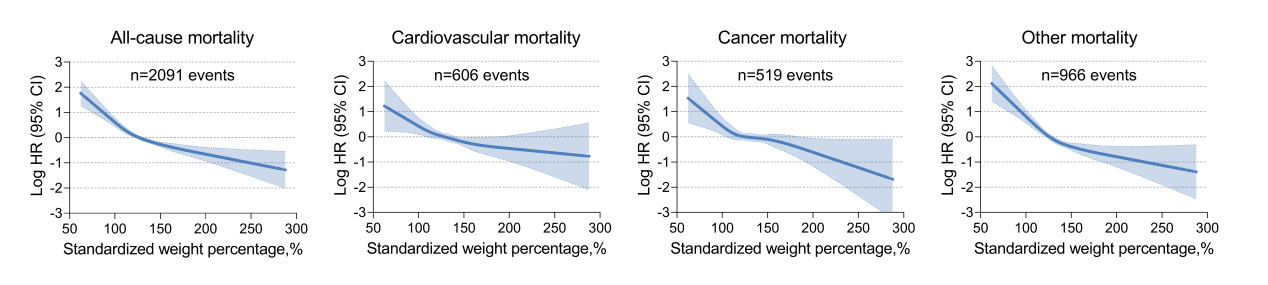
**HRs (solid lines) and 95% CIs (shaded areas) are based on weighted restricted cubic splines. The models were adjusted for age, sex, ethnicity, waist circumference, education, marital status, poverty income ratio, smoking status, alcohol consumption, systolic blood pressure, heart rate, ASCVD, diabetes mellitus, COPD, cancer, aspirin, lipid-lowering drugs, hypoglycemic agents, and laboratory measurements (white blood cell count, hemoglobin, albumin, creatinine, urea nitrogen, glycohemoglobin, total [cholesterol](https://wwwn.cdc.gov/Nchs/Nhanes/2017-2018/BIOPRO_J.htm#LBXSCH), and HDL-C).

**Table S3. Association between other anthropometric indices and mortality**

|  | **All-cause mortality**  HR (95% CI) | **Cardiovascular mortality**  HR (95% CI) | **Cancer mortality**  HR (95% CI) | **Other-cause mortality**  HR (95% CI) |
| --- | --- | --- | --- | --- |
| **Waist circumference (WC)** | | | | |
| Per-10 cm increase | 1.064 (1.063, 1.065) | 1.061 (1.059, 1.063) | 1.001 (0.999, 1.003) | 1.044 (1.043, 1.046) |
| Per-SD increase | 1.109 (1.107, 1.111) | 1.103 (1.099, 1.106) | 1.002 (0.999, 1.004) | 1.075 (1.072, 1.077) |
|  |  |  |  |  |
| **Body roundness index (BRI)** | | | | |
| Per-1 unit increase | 1.190 (1.189, 1.190) | 1.190 (1.188, 1.192) | 0.999 (0.998, 1.001) | 1.261 (1.260, 1.262) |
| Per-SD increase | 1.505 (1.502, 1.508) | 1.506 (1.501, 1.511) | 1.001 (0.997, 1.005) | 1.727 (1.722, 1.731) |
|  |  |  |  |  |
| **Weight-adjusted-waist index (WWI)** | | | | |
| Per-1 unit increase | 1.214 (1.213, 1.216) | 1.277 (1.275, 1.280) | 1.001 (1.000, 1.001) | 1.274 (1.272, 1.276) |
| Per-SD increase | 1.185 (1.184, 1.186) | 1.238 (1.236, 1.240) | 1.000 (0.999, 1.001) | 1.235 (1.234, 1.237) |
|  |  |  |  |  |
| **Relative fat mass (RFM)** | | | | |
| Per-1 unit increase | 1.013 (1.012, 1.013) | 1.040 (1.039, 1.041) | 0.999 (0.996, 1.003) | 1.004 (1.003, 1.004) |
| Per-SD increase | 1.117 (1.114, 1.120) | 1.416 (1.409, 1.424) | 0.997 (0.994, 1.001) | 1.034 (1.030, 1.037) |
|  |  |  |  |  |
| **Body shape index (BSI)** | | | | |
| Per-0.01 increase | 1.262 (1.260, 1.264) | 1.301 (1.297, 1.305) | 1.000 (0.995, 1.006) | 1.318 (1.315, 1.321) |
| Per-SD increase | 1.122 (1.121, 1.123) | 1.139 (1.137, 1.141) | 0.998 (0.996, 1.000) | 1.146 (1.145, 1.148) |
|  |  |  |  |  |
| **Body mass index (BMI)** | | | | |
| Per-5 kg/m^2^ increase | 0.937 (0.937, 0.938) | 0.998 (0.997, 0.998) | 0.925 (0.923, 0.927) | 0.905 (0.904, 0.906) |
| Per-SD increase | 0.915 (0.914, 0.916) | 0.995 (0.994, 0.996) | 0.898 (0.896, 0.901) | 0.872 (0.871, 0.873) |
|  |  |  |  |  |
| **Body surface area (BSA)** | | | | |
| Per-1 unit increase | 0.765 (0.763, 0.767) | 0.952 (0.946, 0.957) | 1.007 (1.000, 1.016) | 0.768 (0.766, 0.770) |
| Per-SD increase | 0.901 (0.900, 0.901) | 0.987 (0.986, 0.989) | 1.003 (0.998, 1.008) | 0.823 (0.822, 0.824) |
|  |  |  |  |  |
| **Standardized weight percentage** | | | | |
| Per-100 increase | 0.724 (0.721, 0.726) | 0.990 (0.083, 0.097) | 0.717 (0.711, 0.723) | 0.618 (0.615, 0.621) |
| Per-SD increase | 0.901 (0.900, 0.902) | 0.995 (0.992, 0.998) | 0.898 (0.895, 0.900) | 0.855 (0.854, 0.857) |

Models were adjusted for age, sex, ethnicity, BMI or waist circumference, education level, marital status, poverty income ratio, smoking status, alcohol consumption, systolic blood pressure, heart rate, ASCVD, diabetes mellitus, COPD, cancer, aspirin, lipid-lowering drugs, hypoglycemic drugs, and laboratory measurements (white blood cell count, hemoglobin, albumin, creatinine, urea nitrogen, glycohemoglobin, total [cholesterol](https://wwwn.cdc.gov/Nchs/Nhanes/2017-2018/BIOPRO_J.htm#LBXSCH), and HDL-C).

**Figure S3. Association of BMI and WHtR with mortality, stratified by age**

**
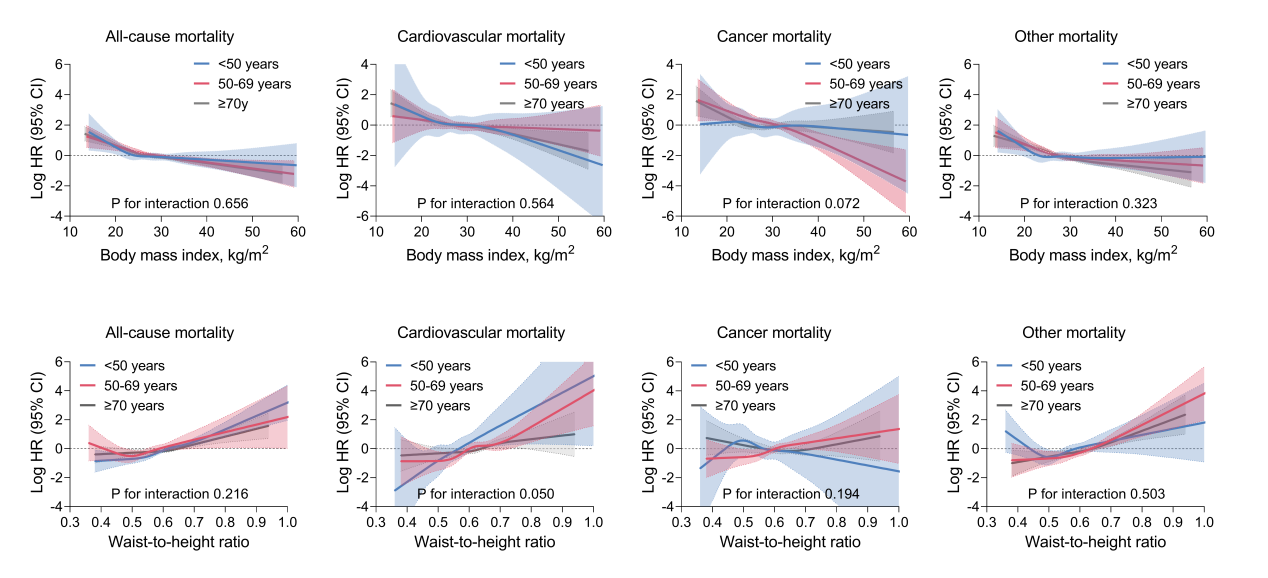
**

Hazard ratios (solid lines) and 95% confidence intervals (shaded areas) are based on weighted restricted cubic splines. The models were adjusted for all predefined covariates **except age**.

**Figure S4. Association of BMI and WHtR with mortality, stratified by sex**


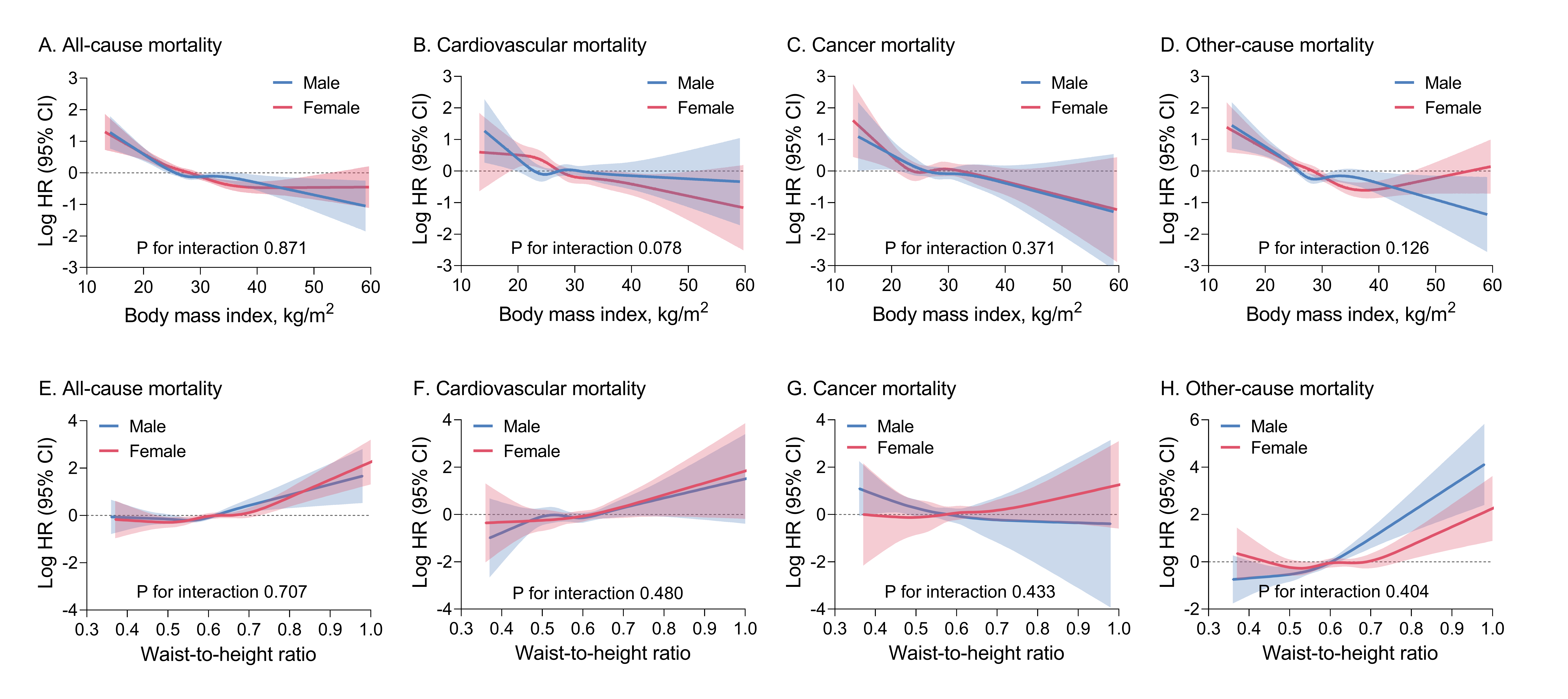
Hazard ratios (solid lines) and 95% confidence intervals (shaded areas) are based on weighted restricted cubic splines. The models were adjusted for all predefined covariates **except sex**.

**Figure S5. Association of BMI and WHtR with mortality, stratified by ethnicity**

**
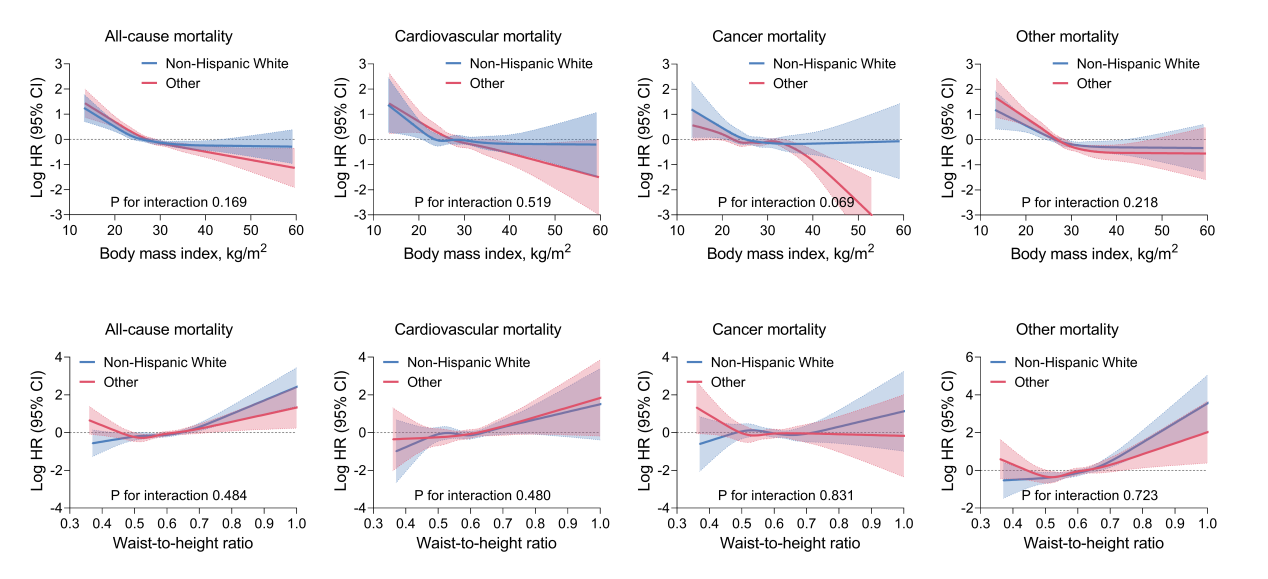
**

Hazard ratios (solid lines) and 95% confidence intervals (shaded areas) are based on weighted restricted cubic splines. The models were adjusted for all predefined covariates **except ethnicity**.

**Figure S6. Association of BMI and WHtR with mortality, stratified by presence of diabetes**

**
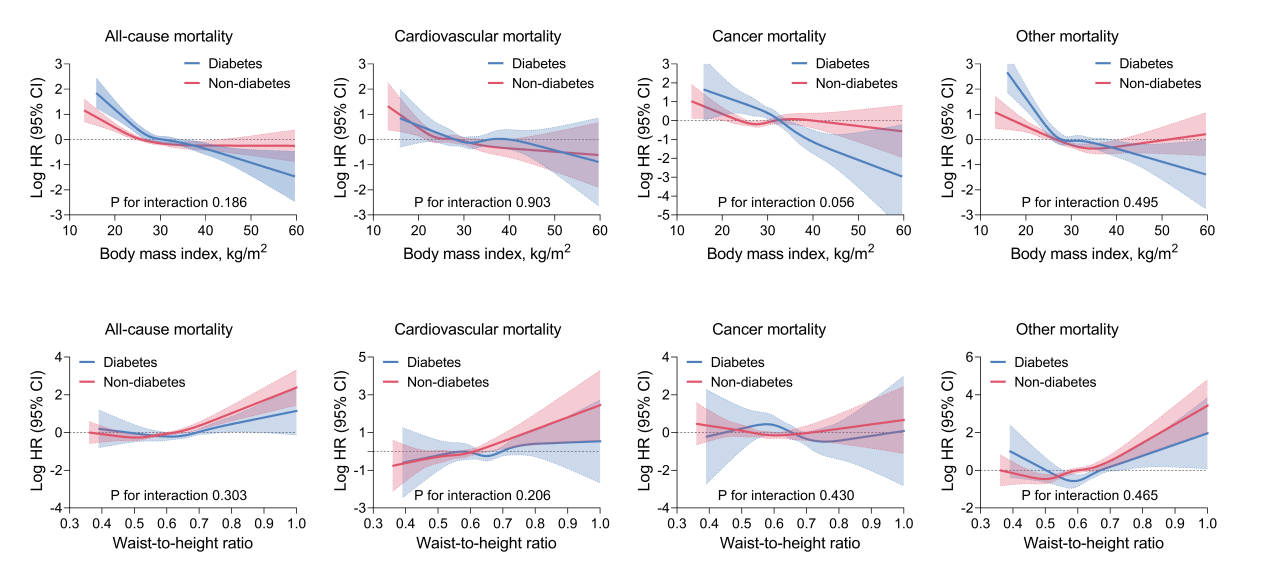
**

Hazard ratios (solid lines) and 95% confidence intervals (shaded areas) are based on weighted restricted cubic splines. The models were adjusted for all predefined covariates **except diabetes**.

**Figure S7. Association of BMI and WHtR with mortality, excluding individuals with less than 1 year of follow-up**

**
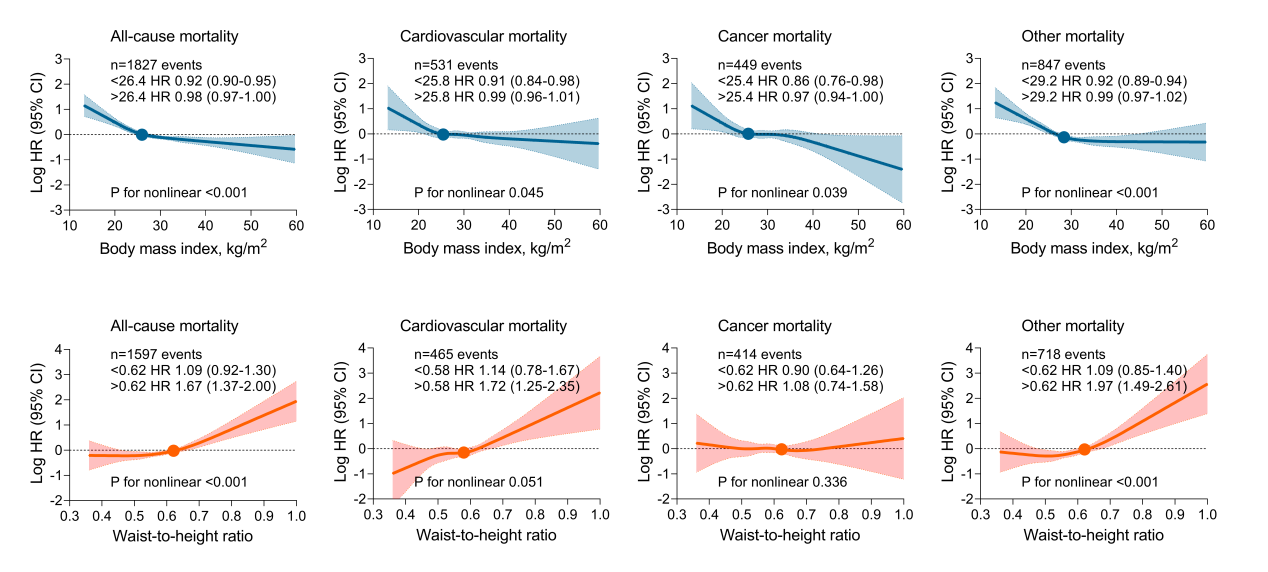
**

Hazard ratios (solid lines) and 95% confidence intervals (shaded areas) are based on weighted restricted cubic splines. The models were adjusted for all predefined covariates, as well as waist circumference for modeling BMI and BMI for modeling WHtR.

**Figure S8. Association of BMI and WHtR with mortality, complete case analysis**


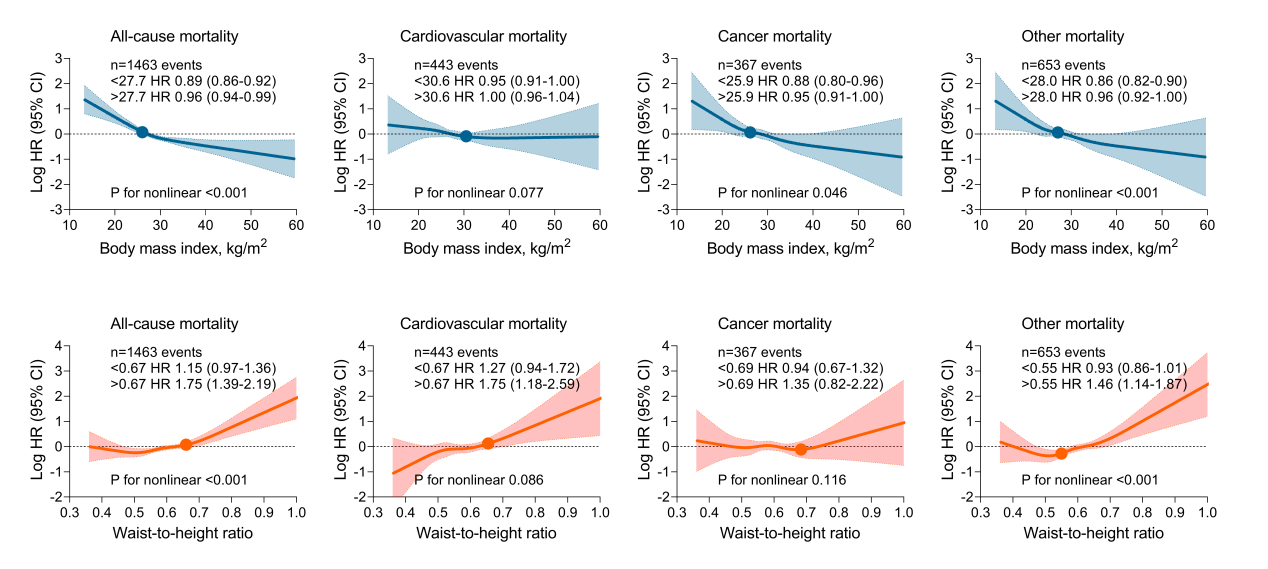


Hazard ratios (solid lines) and 95% confidence intervals (shaded areas) are based on weighted restricted cubic splines. The models were adjusted for all predefined covariates, as well as waist circumference for modeling BMI and BMI for modeling WHtR.
